# Influence of main dietary chemical constituents on the *in vitro* gas and methane production in diets for dairy cows

**DOI:** 10.1186/s40104-016-0109-5

**Published:** 2016-09-17

**Authors:** Laura Maccarana, Mirko Cattani, Franco Tagliapietra, Lucia Bailoni, Stefano Schiavon

**Affiliations:** 1 Department of Comparative Biomedicine and Food Science (BCA), University of Padova, Viale dell’Università 16, 35020 Legnaro (PD), Italy; 2 Department of Agronomy, Food, Natural resources, Animals and Environment (DAFNAE), University of Padova, Viale dell’Università 16, 35020 Legnaro (PD), Italy

**Keywords:** Dairy cows, Dietary manipulation, Gas production, *In vitro* techniques, Methane production

## Abstract

**Background:**

Modification of chemical composition of diets fed to dairy cows might be a good strategy to reduce methane (CH_4_) production in the rumen. Notable reductions of CH_4_ production compared to conventional high-roughages rations were more frequently observed for very concentrated diets or when fat supplements were used. In these cases, the reduction in the gas emission was mainly a consequence of an overall impairment of rumen function with a reduction of fiber digestibility. These strategies do not always comply with feeding standards used in intensive dairy farms and they are usually not applied owing to the risks of negative health and economic consequences.

Thus, the present study evaluated the effects of seven commercial diets with contents of neutral detergent fiber (NDF), protein and lipids ranging 325 to 435 g/kg DM, 115 to 194 g/kg DM, and 26 to 61 g/kg DM, respectively, on *in vitro* degradability, gas (GP), and CH_4_ production.

**Results:**

In this experiment, changes in the dietary content of NDF, crude protein (CP) and lipids were always obtained at the expense or in favor of starch. A decreased of the dietary NDF content increased NDF (NDFd) and true DM (TDMd) degradability, and increased CH_4_ production per g of incubated DM (*P* < 0.001), but not that per g of TDMd. An increase of the dietary CP level did not change *in vitro* NDFd and TDMd, decreased GP per g of incubated DM (*P* < 0.001), but CH_4_ production per g of TDMd was not affected. An increased dietary lipid content reduced NDFd, TDMd, and GP per g of incubated DM, but it had no consequence on CH_4_ production per g of TDMd.

**Conclusions:**

It was concluded that, under commercial conditions, changes in dietary composition would produce small or negligible alterations of CH_4_ production per unit of TDMd, but greater differences in GP and CH_4_ production would be expected when these amounts are expressed per unit of DM intake. The use of TDMd as a standardizing parameter is proposed to account for possible difference in DM intake and productivity.

## Background

Mitigation of methane (CH_4_) production from rumen fermentation represents an important target for animal nutritionists, as also this gas is responsible for global warming. Thus, the manipulation of dietary nutrient composition is often proposed as a strategy that farmers may exploit to reduce the proportion of energy lost by animals as eructated gases (CH_4_) and to improve feed and energy efficiency [1]. There is evidence that the amount of CH_4_ produced in the rumen is influenced by type and content of dietary carbohydrates [2] and lipids [3]. In practice, notable reductions of CH_4_ production compared to conventional high-roughages rations were more frequently observed for very concentrated diets [4] or when fat supplements [3] were used. In these cases the reduction in the gas emission was mainly a consequence of an overall impairment of rumen function with a reduction of fiber digestibility [5]. Thus, these strategies do not always comply with the feeding standards used in intensive dairy farms and they are usually not applied owing to the risks of negative health and economic consequences [6]. Compared to carbohydrates and lipids, minor effects on rumen gas production and methanogenesis are usually attributed to the crude protein (CP). In this regard, [7] observed that CH_4_ production related to CP fermentation was lower compared to that due to carbohydrate fermentation. To date, changes in the dietary CP content have been mainly addressed to reduce feeding costs and N excretion [8, 9]. However, dietary strategies to reduce N excretion could also have an impact because CH_4_ production may decline when dietary CP is replaced by rumen bypass nutrients, including starch, escaping rumen fermentation [10]. Despite this, little information is currently available on the effects on CH_4_ production due to changes in dietary CP content.

This *in vitro* study was aimed at evaluating the extent of alterations of true dry matter degradability (TDMd), total gas (GP) and CH_4_ productions caused by changes in the proportions of the main feed ingredients and of the dietary constituents (structural and non-structural carbohydrates, CP and lipids) in TMR samples representative of rations commonly used in intensive farms in North Italy.

## Methods

### Chemical composition of the diets

Diets used in this experiment were defined after an analysis of a database containing information about ingredient and chemical composition of the rations used by 90 farms considered to be representative of the dairy farm system in North Italy [11, 12].

A corn silage-diet, containing 361, 158, and 33 g/kg of NDF, CP, and lipids, respectively, was used as a reference (Table 1). Six other diets were formulated to produce variations in the proportion of some feed ingredients, and hence in content of a given chemical constituent in favor or at the expense of starchy feeds, with respect to the reference diet. Thus, changes in the dietary content of NDF, CP and lipids, were always obtained at the expense or in favor of starch. Two diets with a low (325 g/kg DM) or a high (435 g/kg DM) content of NDF were formulated by replacing, accordingly, roughages (corn silage, alfalfa hay, and ryegrass hay) with corn and barley grains in the form of meal. The diet with the high NDF content did not contain corn silage, taking into consideration dairy farms that are not allowed to use this feed as they produce milk to be processed as Italian Protected Designation of Origin (PDO) Parmigiano-Reggiano cheese. Other two diets, with a low (115 g/kg DM) or a high CP content (194 g/kg DM), were formulated by replacing, accordingly, soybean meal with cereal grains (corn and barley meal). It must be underlined that the upper level of CP tested in this study corresponded to the maximum value found in the considered database of 90 farms. This value is high if compared with ranges in CP contents (118–186 g/kg DM) reported for rations used in dairy farms of North-Italy [12]. Two diets with different ether extract (EE) content were also formulated. A low EE diet (26 g/kg DM) was achieved by excluding the extruded flaxseed (Linoies, Cortal Extrasoy, Cittadella, Italy), present in the reference diet, and increasing the content of corn, barley and soybean meal. The high EE diet (61 g/kg DM) was achieved by including whole soybean seeds, extruded soybean (Soyfull, Cortal Extrasoy, Cittadella, Italy) and extruded flaxseed. All diets were prepared at the laboratory of the University of Padova. For preparation, about 1 kg of each diet was ground to 1 mm using a hammer mill (Pullerisette 19, Fritsch GmbH, Laborgeratebau, Germany). For each diet, 23 samples were randomly collected, 20 of which were used for the incubations (5 per each of the 4 incubations), whereas the remaining were used for chemical analysis.

**Table 1:** Feed ingredients, chemical composition and gross energy content of seven diets

	Reference	Low NDF	High NDF	Low CP	High CP	Low Lipid	High Lipid
Ingredients, g/kg DM							
Corn silage	351	430	–	375	281	351	351
Alfalfa hay	89	23	134	66	156	89	89
Ryegrass hay	47	–	231	43	52	47	56
Meadow hay	47	–	227	47	52	47	60
Corn grain	205	228	152	258	147	218	160
Barley grain	119	171	92	160	90	122	100
Soybean meal, (sol. extr., 44)	113	119	126	27	188	126	18
Whole soybean seeds, cracked	–	–	–	–	–	–	68
Extruded soybean seeds	–	–	–	–	–	–	68
Extruded flaxseed seeds	29	29	38	24	34	–	29
Chemical composition, g/kg DM							
Crude protein (CP)	158	152	161	115	194	161	158
Starch	273	273	100	332	176	265	233
NDF	361	325	435	358	357	359	360
Hemicellulose	169	171	189	172	158	171	169
ADF	192	154	246	186	199	188	191
Cellulose	163	134	203	160	167	163	163
Lignin_(sa)_	29	20	43	26	32	25	28
NFC^a^	395	443	302	446	357	402	367
Ether extract	33	34	38	34	34	26	61
Ash	53	46	64	47	58	52	54
Gross energy, MJ/kg DM^b^	16.8	16.9	17.3	16.8	17.3	16.5	16.7

^a^ *NFC* Not Fiber Carbohydrates computed as 100 - NDF - CP - EE - Ash

^b^Measured by a bomb calorimeter method [16]

Diets were analyzed in triplicate for dry matter (DM: # 934.01; [13]), nitrogen (# 976.05; [13]), EE (# 920.29; [13]), and ash (# 942.05; [13]). Neutral detergent fibre (NDF), inclusive of residual ash, was determined with α-amylase using the Ankom^220^ Fibre Analyzer (Ankom Technology, NY, USA). Acid detergent fibre (ADF), inclusive of residual ash, and sulphuric acid lignin (lignin_(sa)_) were determined sequentially after NDF determination [14]. Starch content was determined after hydrolysis to glucose [13] by liquid chromatography [15]. Gross energy content of diets (MJ/kg DM) was determined in duplicate by a bomb calorimeter method [16].

### Incubation

The 7 diets were incubated in 4 repeated incubation runs, conducted in 4 successive wk. Two incubations were stopped at 24 h, whereas the other two were stopped after 48 h. The incubation times of 24 and 48 h were chosen as they are, respectively, the reference times for measuring *in vitro* GP [17] and *in vitro* degradability of NDF [18]. In each of the four incubation runs, we tested 7 diets × 5 replications, plus 5 blanks (bottles containing only the buffered rumen fluid; 5 blanks/run), for a total of 160 bottles incubated. A commercial GP apparatus (Ankom^RF^ Gas Production System, Ankom Technology®, NY, USA) was used, consisting of 40 bottles equipped with pressure sensors (pressure range: from - 69 to 3,447 kPa; resolution: 0.27 kPa; accuracy: ± 0.1 % of measured value) and wireless connected to a computer. Each bottle (317 mL) was filled with 1.000 ± 0.0010 g of diet, 100 mL of a buffer solution, and 50 mL of rumen fluid (headspace volume = 167 mL), keeping the headspace of bottles flushed with CO_2_.

The buffer solution was prepared according to [17], heated in a water bath at 39 ± 0.4 °C and purged continuously with CO_2_ for 30 min, to maintain anaerobic conditions. Rumen fluid was collected by an esophageal probe, as described by [19], 2 h before morning feeding from 3 dry Holstein-Friesian cows housed at the experimental farm of the University of Padova (Italy) and fed hay *ad libitum* and 2.5 kg/d of concentrates (0.5 kg of dry sugar beet pulp, 1 kg of corn grain, and 1 kg of sunflower meal). During the collection of rumen fluid, cows were handled according to the Italian law on animal care (Legislative Decree No. 26 of March 14, 2014). Rumen fluid was poured into thermal flasks preheated to 39 ± 0.5 °C, immediately transferred to the laboratory, strained through 3 layers of cheesecloth, to eliminate feed particles, and mixed with buffer solution in a 1 to 2 ratio [17]. Operations were conducted under anaerobic conditions, by flushing with CO_2_, and required less than 30 min to be completed. Bottles were placed in a ventilated oven at 39 ± 0.4 °C and automatically vented at a fixed pressure (6.8 kPa), to avoid overpressure conditions and alterations of gas and CH_4_ measures [20]. *In vitro* GP was monitored in continuous, using a setting of the GP system that allows to record pressure values every minute. Other *in vitro* parameters (rumen degradability, VFA and N-NH_3_ concentrations, CH_4_ production) were measured only at the end of incubation (at 24 or 48 h), to avoid opening of the oven during the incubation, with alteration of fermentation process.

At the end of incubations (24 or 48 h), two aliquots (5 mL) of fermentation fluid were collected from each bottle and stored at −20 °C with 1 mL of metaphosphoric acid (25 %, w/v) to be later analyzed for ammonia N and volatile fatty acids (VFA). The content of ammonia N was measured using the FIAstar™ 5000 Analyzer (FOSS Analytical, Hilleroed, Denmark). The VFA profile was analyzed by GC with flame ionization detection (7820A GC system, Agilent Technologies, Milan, Italy) using a 30-m stainless steel column (J&W DB-FFAP, Agilent Technologies, Milan, Italy) and H_2_ as carrier gas (flow rate: 30 mL/min; isothermal oven temperature: 150 °C). Fermentation fluids were filtered into weighed crucibles (30 mL, Robu Glasfilter-Geräte GMBH®, Hattert, Germany) and analyzed for residual NDF using a Fibretech Analyzer (VELP® Scientifica, Milan, Italy).

At the end of each incubation (24 or 48 h), gas was collected with a 10-mL gas-tight syringe (Artsana S.p.A., Como, Italy) from the bottle headspace (HS). At each sampling, the syringe was flushed in order to collect a homogeneous sample, which was immediately transferred into a 9-mL vacuette (Greiner Bio-One GmbH, Kremsmunster, Austria). From each vacuette, an aliquot (10 μL) of gas was sampled with a gas-tight syringe (1701 N, Hamilton, Bonaduz, Switzerland) and immediately analyzed for CH_4_ concentration by GC with flame ionization detection (7820A GC system, Agilent Technologies, Milan, Italy) using a 15-m carbon layer column (GS-CarbonPLOT, Agilent Technologies, Milan, Italy) and H_2_ as carrier gas (flow rate: 1.6 mL/min; isothermal oven temperature: 40 °C). An 11-point calibration curve was generated from eleven gas mixtures containing 2, 4, 8, 16, 24, 32, 60, 100, 140, 180, and 240 mL of CH_4_/L (99.5 % pure, SAPIO s.r.l., Monza, Italy), respectively, and known volumes of air. Mixtures were prepared using the same graduated gas-tight syringe (1701 N, Hamilton). The calibration regression had R^2^ > 0.99.

### Computations

The NDF degradability (NDFd) and the true DM degradability (TDMd) were calculated according to [18].

Recently, [20], using vented bottles connected to tight bags for gas collection, calculated CH_4_ production (mL) as: [CH_4_ concentration in HS] × [HS volume] + [CH_4_ concentration in the gas bag × GP]. To evaluate the possibility of avoiding the use of bags, to save space and increase the number of replicates, amount of CH_4_ lost with gas venting was computed using the unpublished data of a previous study, where 4 forages and 3 concentrates were incubated in 42 bottles (6 bottles/feed) for 6, 24, or 48 h using the same GP equipment and the same operative conditions of the present experiment. It was found that total CH_4_ production is predictable, with acceptable precision and accuracy, as: − 0.0064 × [CH_4_ concentration in HS × (HS volume + GP)]^2^ + 0.9835 × [CH_4_ concentration in HS × (HS volume + GP)]. This equation had a residual standard deviation of only 0.1770 mL, and R^2^ = 0.9993. Thus, the present experiment was conducted without the use of tight bags for gas collection. The CH_4_ production was computed using the above described equation and it was expressed as mL/g DM incubated, mL/g of digested NDF (dNDF), mL/g TDMd, or mL/100 mL GP. *In vitro* GP and CH_4_ were also predicted from VFA production, according to [21].

### Statistical analysis

The mean of the 5 replications for each diet in each incubation run was computed. These 28 means were analyzed using PROC MIXED of SAS [22] using a model considering the diet (D; 6 df), the incubation time (IT, 1 df), and the interaction diet × incubation time as fixed factors, the run within incubation time (2 df) as a random blocking factor and the residual error term *e* (18 df). As the diet × incubation time interaction was never significant, it was excluded from the model. Contrasts were run to analyze statistical differences among diets with different contents of a given chemical constituent, using the Bonferroni adjustment to perform multiple comparisons.

## Results

Changes of the feed ingredients proportions and of dietary contents of chemical constituents had influence on the various parameters of *in vitro* fermentation (Table 2). As expected, pH values measured at the end of fermentation were not influenced by the dietary changes. The ammonia N content increased with increasing dietary CP content (*P* = 0.014). No influence of the diets was observed on the VFA production, but the proportion of acetate or butyrate decreased (*P* < 0.001) or increased (*P* = 0.004), respectively, with a decrease of NDF content, whereas the proportion of propionate decreased (*P* = 0.048) with increasing dietary CP. Thus, the ratio between acetate and propionate decreased with decreasing level of NDF (*P* = 0.001) and of CP (*P* < 0.001), and the corresponding increasing level of starch. Increasing proportions of dietary CP increased the proportion of other VFA (*P* < 0.001) found in the rumen fluid. Changes of dietary EE content had no consequence on the various rumen fluid parameters. The prolongation of the incubation time from 24 to 48 h increased the VFA production (*P* = 0.029), but it did not influence pH and ammonia N values.

**Table 2:** Effect of diets and incubation time on pH, ammonia N concentration, volatile fatty acid production (VFA) and molar proportions of acetate, propionate, butyrate and other VFA

	pH	Ammonia N, mg/L	VFA, mmoL/L	Acetate (Ac), % VFA	Propionate (Pr), % VFA	Ac:Pr	Butyrate, % VFA	Other VFA, % VFA
Diet								
Reference	6.87	202	5.08	56.1	23.5	2.40	16.0	4.47
Low NDF	6.88	192	5.37	55.8	23.7	2.36	16.2	4.29
High NDF	6.90	223^A^	4.44	58.5^A^	23.1	2.53	13.3^B^	4.26^B^
Low CP	6.85	171	5.06	56.0	24.3	2.31	16.1	3.71^B^
High CP	6.87	222^A^	4.89	57.2	22.8	2.51	14.6	4.85^A^
Low lipid	6.87	207	4.96	56.4	23.7	2.39	15.5	4.26^B^
High lipid	6.90	197	4.99	56.7	23.5	2.41	15.4	4.36
SEM	0.043	28.5	0.231	0.25	0.64	0.062	0.91	0.158
*P*-value	0.46	0.008	0.25	<0.001	0.06	0.001	0.002	<0.001
*P*-value of contrasts								
Low vs High NDF	0.99	0.44	0.23	<0.001	0.99	0.001	0.004	0.99
Low vs High CP	0.99	0.014	0.99	0.048	0.048	<0.001	0.99	<0.001
Low vs High lipid	0.99	0.99	0.99	0.99	0.99	0.65	0.99	0.99
Incubation time								
24 h	6.95	202	4.47	57.2	22.9	2.50	15.8	3.87
48 h	6.80	202	5.47	56.1	24.1	2.33	14.7	4.76
SEM	0.055	38.7	0.123	0.13	0.82	0.077	1.15	0.201
*P*-value	0.31	0.99	0.029	0.026	0.39	0.26	0.58	0.09
RMSE	0.032	17.5	0.462	0.49	0.60	0.112	0.87	0.147

Values with different superscripts within column are significantly (*P* < 0.05) higher (A) or lower (B) compared to the reference diet (containing 361, 158, and 33 g/kg DM of NDF, CP, and lipids)

*Low NDF* low NDF diet (325 g/kg DM); *High NDF* high NDF diet (435 g/kg DM), *Low CP* low protein diet (CP, 115 g/kg DM), *High CP* high protein diet (CP, 194 g/kg DM), *Low lipid* low lipid diet (EE, 26 g/kg DM), *High lipid* high lipid diet (EE, 61 g/kg DM)

The NDFd, TDMd, and the GP expressed per unit of incubated DM or per unit of TDMd (Table 3) increased with a decrease of NDF content (*P* < 0.001 for all). When the NDF content decreased, the CH_4_ production increased per unit of incubated DM (*P* < 0.001) and per g of digested NDF (*P* = 0.002), but not per unit of TDMd. The increased dietary CP content, with the corresponding decrease of starch, had no influence on NDFd or TDMd, but GP was lowered. No influence was observed on the production of CH_4_, except when this was expressed as a proportion of GP (mL CH_4_/100 mL GP; *P* < 0.001). An increased inclusion of extruded oilseeds in the diet reduced both NDFd (*P* = 0.003) and TDMd (*P* = 0.028), and the measured GP expressed per g of incubated DM (*P* = 0.017), but no influences were observed on the CH_4_ yield. A prolonged duration of the incubation, from 24 to 48 h, increased NDFd (*P* = 0.009), TDMd (*P* = 0.007), and CH_4_ production, both per unit of incubated DM (*P* = 0.014) and per unit of TDMd (*P* = 0.027). The correlation between measured and predicted values showed R^2^ to be 0.78 and 0.74, respectively, for gas and CH_4_ measures (data not shown), and the relationship obtained by regressing measured values of CH_4_ (mL/g DM; y) against those predicted (mL/g DM; x) was the following: measured CH_4_ = 0.95 × predicted CH_4_ – 2.6. Predicted values of GP and CH_4_ productions were not influenced either by dietary changes or by incubation time.

**Table 3:** Effects of diets and incubation time on in vitro degradability of NDF (NDFd) and of true DM (TDMd), gas production (GP) and methane (CH_4_) production, and predicted values of GP and CH_4_ production

	Degradability		Actual GP, ml per:			Actual CH_4_ mL per			Predicted GP	Predicted CH_4_
	NDFd, g/kg NDF	TDMd, g/kg DM	g DM	g TDMd	g DM	g dNDF	g TDMd	100 mL GP	mL/g DM	mL/g DM
Diet										
Reference	567	840	274	325	34.2	168	40.8	12.5	244	38.8
Low NDF	612^A^	872^A^	288^A^	331	34.4	174	39.3	11.9	250	39.6
High NDF	480^B^	767^B^	228^B^	300^B^	30.8^B^	151^B^	39.9	13.5^A^	220	35.9
Low CP	545	835	277	333	33.4	169	39.9	12.0	242	38.0
High CP	542	831	255^B^	310	34.0	176	41.0	13.3^A^	234	37.8
Low lipid	583	845	265	315	33.1	159	39.0	12.3	251	37.2
High lipid	531	826	253^B^	304^B^	32.2^B^	169	38.8	12.5	238	37.9
SEM	10.3	4.2	5.6	7.2	0.53	3.4	0.63	0.40	10.9	2.09
*P*-value	<0.001	<0.001	<0.001	<0.001	<0.001	<0.001	0.025	<0.001	0.33	0.70
*P*-value of contrasts										
Low vs High NDF	<0.001	<0.001	<0.001	<0.001	<0.001	0.002	0.99	<0.001	0.99	0.99
Low vs High CP	0.99	0.99	<0.001	0.009	0.99	0.99	0.99	<0.001	0.99	0.99
Low vs High lipid	0.003	0.028	0.017	0.99	0.99	0.99	0.99	0.99	0.99	0.99
Incubation time										
24 h	472	801	250	314	29.6	172	37.0	11.9	214	34.9
48 h	631	860	276	320	36.7	161	42.6	13.2	260	41.5
SEM	10.5	3.5	7.4	8.8	0.59	1.8	0.66	0.54	7.6	1.69
*P*-value	0.009	0.007	0.14	0.69	0.014	0.046	0.027	0.22	0.06	0.12
RMSE	15.5	7.2	4.0	7.6	0.69	3.2	0.94	0.25	21.9	3.78

Values with different superscripts within column are significantly (*P* < 0.05) higher (A) or lower (B) compared to the reference diet (containing 361, 158, and 33 g/kg DM of NDF, CP, and lipids)

*Low NDF* low NDF diet (325 g/kg DM), *High NDF* high NDF diet (435 g/kg DM), *Low CP* low protein diet (CP, 115 g/kg DM), *High CP* high protein diet (CP, 194 g/kg DM), *Low lipid* low lipid diet (EE, 26 g/kg DM), *High lipid* high lipid diet (EE, 61 g/kg DM)

## Discussion

### General considerations

The diets were formulated using feed ingredients commonly used in the Po valley (North-East of Italy) and composed mainly by cereal grains, corn silage and various hays. It should be preliminarily considered that in this study the variation in the content of the various nutrients, namely, NDF, CP and lipid, were always achieved by decreasing or increasing the proportion of dietary starch. In the scientific literature, *in vitro* evaluation of gas and CH_4_ productions is commonly carried out using single feeds, mainly forages, whereas less information is available for complete diets [23]. *In vivo* measurement of gas and CH_4_ production requires expensive equipment and it is labor and time consuming. *In vitro* techniques would permit a much more simple determination of the dietary characteristics which can influence the potential emission of gas and CH_4_ from their fermentation in a simulated rumen environment [20]. Studies of the relationships between *in vitro* and *in vivo* gas and CH_4_ productions are lacking [23]. However, a recent study suggested that *in vitro* gas and CH_4_ measurements can be indicative of the trend of *in vivo* CH_4_ production originating from different combinations of feed ingredients [24]. This study was aimed at evaluating if changes in the diet composition might or might not have notable influence on gas and CH_4_ productions.

After 24 h of fermentation, the measured GP of the various diets was, on average, 250 mL/g DM, suggesting that a cow consuming 20 kg/d DM might produce about 5,000 L/d of gas. The CH_4_ production from fermentation of these diets ranged 30.8 to 34.4 mL/g DM, suggesting that, for a DM intake of 20 kg/d, a cow might produce 616 to 688 L/d of CH_4_. Sauer et al. (1998) reported *in vivo* CH_4_ production of lactating cows in the order of 622 L/d, corresponding to 38.9 mL/g DM intake. In the study of [25], CH_4_ production from dairy cows was 29.2 mL/g DM intake, whereas [26] reported for sheep an averaged CH_4_ production of 31.0 mL/g DM intake. In a continuous culture fermenter, [27] measured an averaged CH_4_ production of 33.0 mL/g DM. The *in vitro* CH_4_ production of the dairy rations tested by [23] varied from 30.1 to 35.9 mL/g DM, a range consistent with results obtained in this study. However, comparison with data from literature is difficult because huge variations in gas and CH_4_ productions are commonly observed across experiments, even for diets with similar composition. This is the consequence of a combination of different biological, as rumen fluid characteristics, and technical factors, as fermentation procedures and equipment for collection, measurement and analysis [28]. In this experiment the observed GP and CH_4_ were regressed against the GP and CH_4_ values predicted from the stoichiometry of the VFA production, to obtain an internal control of consistency of the data. The correlations found (R^2^ = 0.78 and 0.74, respectively, for gas and CH_4_ measures) are acceptable considering the rather narrow range of variation in GP and CH_4_ production caused by the dietary changes. It was observed that the SEM of predicted GP and CH_4_ production (21.9 and 3.78 mL/g DM, respectively) was about five times greater than the corresponding values of the measured GP and CH_4_ production. In other words, the measure of the VFA production was less precise than the GP and CH_4_ measures.

### Effects on NDF degradability, gas and CH_4_ production due to changes in the NDF content

In this experiment, the diet with the low level of NDF (325 g/kg DM) was based on corn silage and a small amount of alfalfa hay (23 g/kg DM) as forage sources. On the contrary, the diet with the high level of NDF (435 g/kg DM) was based on different hays and did not contain corn silage. This is of interest in Italy as use of silages for the production of important Protected Denomination of Origin (PDO) cheeses (i.e., Parmigiano-Reggiano) is forbidden by specific feeding regulations. From the results it emerged that NDF degradability was negatively influenced by the dietary NDF content, partially because of the complete replacement of corn silage with hays. This would be in agreement with previous observations that the NDF fraction of hay samples collected in the same area of the present experiment were less degradable than corn silage samples [19].

In diets similar for ingredient composition to those used in this experiment, [12] observed an increased CH_4_ production (35.6 to 44.3 mL/g DM) with decreasing NDF content. In the present study, a decrease from 435 to 325 g NDF/kg DM increased CH_4_ production from 30.8 to 34.4 mL/g DM (+11.6 %). This is consistent with an increased true degradability of the feed (+14 %), but also of the NDF fibrous fraction (+27 %). This seems to be contradictory with current literature, as a decrease of dietary NDF commonly reduces the NDF degradability [12]. However, also [29] evidenced that a decrease of the dietary NDF content increased by 43 % *in vivo* CH_4_ production expressed per unit of dNDF. Results also evidenced that GP increased with a decrease of NDF content even when expressed per unit of TDMd (+10 %), but no influences were observed on the CH_4_ production per unit of TDMd. A different trend emerged when CH_4_ production was referred to the total GP. In this case, the decrease of NDF lowered the CH_4_ proportion by about 12 % (13.5 to 11.9 mL CH_4_/100 mL GP, for the high and the low content of NDF, respectively). Results suggest that a decrease of dietary NDF content, achieved from a complete replacement of hays with corn silage and cereal grains, might increase feed digestibility without changing GP and CH_4_ produced per unit of digested material.

### Effects on gas and CH_4_ production due to changes in the CP content

In this experiment an increased proportion of CP, in replacement of starch, caused a reduction of GP. The negative influence of dietary CP on GP has been observed by others in the past. Such an effect was attributed to the buffer capacity of CP, that reduces the indirect CO_2_ released from the buffered rumen fluid, and to the stoichiometry of protein fermentation, that differs from that of carbohydrates [30, 31]. In this study, increase of the CP content was associated to a decrease of dietary starch. As a consequence, some VFA as iso-butyric and iso-valeric acids were increased, being end-products of protein degradation [32], whereas the production of propionate decreased and the ratio between acetate and propionate increased. Changes in the dietary CP proportion had no effect on CH_4_ production when expressed both per unit of incubated DM and per unit of TDMd. Thus, as CP depresses GP, an increased proportion of CP increases the CH_4_ concentration on total GP.

### Effects on gas and CH_4_ production due to changes in the lipid content

In this experiment changes in the dietary fat content of the diets were achieved by changing the proportions of extruded flaxseeds, extruded soybean and whole soybean seeds. The threshold of 60 g fat/kg DM was considered to be the upper limit to avoid a possible impairment of feed digestibility [6, 33].

The effect of dietary lipids on CH_4_ production is dependent on the source, FA profile, level of inclusion, and diet composition [34]. The level of supplementation and the physical form of the lipid supplement affect its availability in the rumen, and these factors appear to be more important than the FA profile [35]. In this regard, [36], using dairy cows housed in respiratory chambers, found that, compared to the control, the average reduction in CH_4_ (L/kg DMI) per 10 g/kg of crude fat added was persistent throughout lactation. The same authors observed that the most effective lipid source in reducing methanogenesis was a commercial vegetal rumen protected fat fortified with hydroxy-methionine-analog-isobutyrate (−5.5 % of CH_4_), followed by vegetal rumen protected fat (−2.3 %), and by whole cracked rapeseed (−0.8 %). In the experiment of [35], only crushed canola seeds lowered CH_4_ production per unit of digestible DM intake (−15 %), whereas crushed flaxseeds and crushed sunflower seeds did not reduce CH_4_ production compared to the control diet (a diet supplemented with a commercial source of calcium salts of long chain fatty acids). In the current experiment, fat addition reduced feed degradability, particularly that of the NDF fraction. No influences were found on total VFA production and on the proportion of acetate, propionate and butyrate, whereas GP and CH_4_ productions decreased by 8 and 6 %, respectively, compared to the reference diet. However, differences were greatly reduced when GP and CH_4_ were expressed per unit of TDMd suggesting that, under the commercial conditions evaluated in this study, small reductions of CH_4_ might be achieved.

## Conclusions

Changes of the ingredient and chemical composition of diets were analyzed to evaluate benefits in the amount of CH_4_ produced, for the north eastern Italian dairy production chain. It was found that a replacement of hays with corn silage and cereals might increase GP and CH_4_ per unit of DM intake. An increase of the dietary CP content would reduce GP with no influences in the amount of CH_4_ produced, whereas a moderate addition of cracked soybean seeds, and extruded flaxseed had few, or any, influence on the *in vitro* GP and CH_4_ productions. In general, none of the various strategies tested in the present work was able to reduce the amount of CH_4_ produced, especially if this production is expressed per unit of digestible DM intake. More research is needed to evaluate the effectiveness of strategies to reduce the CH_4_ emissions, and relationships between *in vitro* and *in vivo* gas and CH_4_ productions need to be developed.

## Abbreviations

ADF, Acid detergent fibre expressed inclusive of residual ash; CP, Crude protein; DM, Dry matter; EE, Ether extract; GP, Gas production; NDF, Neutral detergent fibre assayed with a heat stable amylase and expressed inclusive of residual ash; NDFd, NDF degradability; lignin(sa), Lignin determined by solubilization of cellulose with sulphuric acid; NRC, National Research Council; RMSE, Root mean square error; SEM, Standard error of the mean; TDMd, True DM degradability; VFA, Volatile fatty acids
